# Association between malnutrition and stroke-associated pneumonia in patients with ischemic stroke

**DOI:** 10.1186/s12883-023-03340-1

**Published:** 2023-08-03

**Authors:** Dongze Li, Yi Liu, Yu Jia, Jing Yu, Fanghui Li, Hong Li, Lei Ye, Xiaoyang Liao, Zhi Wan, Zhi Zeng, Yu Cao

**Affiliations:** 1 West China School of Nursing, Sichuan University/ Department of Emergency Medicine, West China School of Medicine, West China Hospital, Sichuan University, Chengdu, China; 2 Laboratory of Emergency Medicine, Disaster Medical Center, West China School of Medicine, West China Hospital, Sichuan University, Chengdu, China; 3 Department of General Practice, General Practice Medical Centre, West China School of Medicine, West China Hospital, Sichuan University, Chengdu, China; 4 Department of Emergency Medicine, West China School of Medicine, West China Hospital, Sichuan University, 37 Guoxue Road, Chengdu, Sichuan, 610041 China

**Keywords:** Malnutrition, Stroke-associated pneumonia, Ischemic stroke, Nutrition assessment

## Abstract

**Background:**

Malnutrition is associated with a high risk of mortality in adults with ischemic stroke (IS). This study aimed to investigate the relationship between malnutrition and the risk of stroke-associated pneumonia (SAP) as only a few studies examined the relationship between malnutrition and the risk of SAP in IS.

**Methods:**

Patients were included from emergency departments of five tertiary hospitals in the REtrospective Multicenter study for Ischemic Stroke Evaluation (REMISE) study from January 2020 to December 2020. Malnutrition was defined according to the Controlling Nutritional Status (CONUT), Geriatric Nutritional Risk Index (GNRI), and Prognostic Nutritional Index (PNI) systems. Multivariable logistic regression analysis was used to explore the association between malnutrition and risk of SAP.

**Results:**

We enrolled 915 patients with IS, 193 (14.75%), 495 (54.1%), and 148 (16.2%) of whom were malnourished according to the PNI, CONUT, and GNRI scores, respectively. SAP occurred in 294 (32.1%) patients. After adjusting for confounding influencing factors in the logistic regression analysis, malnutrition (moderate and severe risk vs. absent malnutrition) was independently associated with an increased risk of SAP based on the PNI (odds ratio [OR], 5.038; 95% confidence interval [CI] 2.435–10.421, *P* < 0.001), CONUT (OR, 6.941; 95% CI 3.034–15.878, *P* < 0.001), and GNRI (OR, 2.007; 95% CI 1.186–3.119, *P* = 0.005) scores. Furthermore, adding malnutrition assessment indices to the A^2^DS^2^ score significantly improved the ability to predict SAP by analysis of receiver operating characteristic curves and net reclassification improvement.

**Conclusion:**

Malnutrition was notably prevalent in patients with IS and independently associated with an increased risk of SAP. Further studies are required to identify the effect of interventions on malnutrition to reduce the risk of SAP.

**Supplementary Information:**

The online version contains supplementary material available at 10.1186/s12883-023-03340-1.

## Introduction

Stroke is a main cause of death and long-term disability globally, and stroke-associated pneumonia (SAP) is a common and serious consequence of ischemic stroke (IS), affecting 7% to 38% IS patients [1–3]. Previous studies have indicated that SAP worsens adverse clinical outcomes and increases the risk of severe disability and mortality [1, 4–6]. Moreover, it prolongs the duration of hospitalization and increases the treatment costs for IS in hospitals [3, 7, 8]. Therefore, clarifying risk factors for SAP may play an important role in preventing SAP in patients with IS.

Attempts have been made to determine the risk of SAP in IS patients using various risk variables and predictive scores. Some studies indicated that age, dysphagia, atrial fibrillation, severity of the stroke, and stroke-induced immunodepression syndrome were linked to the risk of SAP, and certain inflammatory indicators, including neutrophil to lymphocyte ratio (NLR), C-reactive protein, and procalcitonin, had predictive value for SAP in IS patients; however, no single marker or pattern has exhibited sufficient accuracy and reliability [1, 2, 5, 9–13]. Therefore, some risk stratification scoring systems, including the Friedant Pneumonia Predict Score, A^2^DS^2^ Score, Kwon Pneumonia Score, PASS Pneumonia Rule, and ISAN Score, have been derived to predict SAP events using some routinely available clinical risk variables [2, 3, 13]. Of these scoring systems, information on the clinical background and severity of stroke was enrolled, resulting in the identification of patients who would develop SAP, with limited calibration and discrimination. Therefore, it may be necessary to obtain more dimensional information to evaluate the risk of SAP in IS patients.

Malnutrition, as screening by the Geriatric Nutrition Risk Index (GNRI), Controlling Nutritional Status (CONUT), and Prognostic Nutritional Index (PNI) scores at admission, is prevalent in adults with IS and is linked to an increased risk of all-cause mortality and ineffective functional consequences. Moreover, Patients with IS are especially vulnerable to malnutrition due to age, dysphagia, immunodepression, cognitive deficits, and even limited mobility [14]. It suggested that pre-existent malnutrition may be worse under attack a IS incident, which may result in patients being placed at high risk of adverse outcomes. Recent researches have indicated that early nutrition therapy utilizing early enteral nutrition combined with probiotics can successfully enhance the nutritional condition of patients with stroke, reduce systemic inflammatory levels, and minimize the incidence of infection [15]. Therefore, malnutrition may be an intervention target to reduce the risk of SAP, and information from early malnutrition evaluations may be used to identify IS patients who are at high risk of SAP. However, a few research demonstrated the usefulness of malnutrition assessment tool, such as GNRI, PNI, and CONUT, to identify the risk of SAP in IS patients [16, 17]**.** Therefore, the purpose of this research was to investigate whether IS patients with worse malnutrition, assessed by GNRI, PNI, and CONUT, had a higher risk of SAP, and whether these tools provided an additional value beyond traditional tools for predicting the incidence of SAP.

## Methods

### Study design

The REtrospective Multicenter study for Ischemic Stroke Evaluation (REMISE) study was a multicenter retrospective cohort study, registered at Chinese Clinical Trial Registry (Identifier: ChiCTR2100052025). Patients with IS were recruited from emergency departments of five tertiary hospitals in Sichuan, China from January 2020 to December 2020. Regarding the COVID-19 pandemics, patients without negative nucleic acid test for SARS-CoV-2 within 24 h are temporarily placed in isolation wards. There are only a small number of COVID-19 confirmed patients in Sichuan Province, and none of the IS patients during this study periods were infected. This study adhered to the Declaration of Helsinki and was approved by the ethics committee of West China Hospital of Sichuan University. Retrospective chart review without need of informed consent form (Approved No. of ethic committee: 2021–1175).

### Study population

**Fig. 1:**
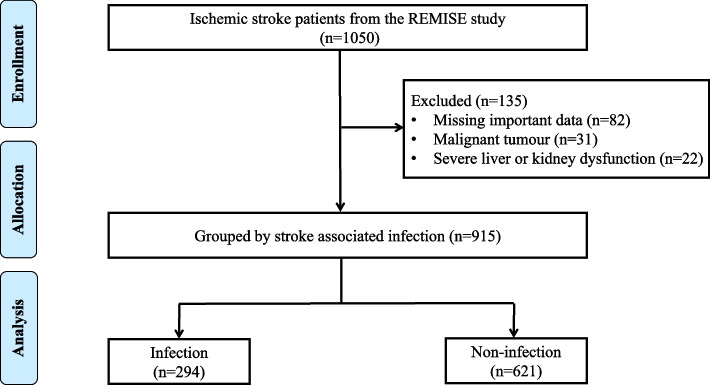
Flow chart of the enrollment of participants in this study

We investigated the association between malnutrition and the risk of SAP in individuals with IS. Patients were included if they were first diagnosed with IS according to the 2019 American Heart Association Stroke guidelines and did not receive mechanical ventilation within 7 days of stroke onset [18]. We excluded individuals with a diagnosis of subarachnoid hemorrhage or transient ischemic attack, combined with malignant tumors, severe liver or kidney dysfunction, a history of clinical signs of infection on admission or 30 days before IS onset, or unavailable data to obtain PNI, CONUT, or GNRI scores on admission. A diagram illustrating the individuals selection process is shown in Fig. 1.

Sample Size Calculation: assuming that the AUROC of three malnutrition assessment tools for predicting SAP is greater than 0.75. And the smallest ratio of sample sizes in non-malnutrition to malnutrition groups is 6. To satisfy that Type I error (α, significant) and Type II error (β, 1-power) should be less than 0.01, the smallest sample size was 252.

### Data collection and measures

Information was gathered from the REMISE database. Using standard case report forms, trained physicians collected baseline data, including demographic and characteristic information, vital signs, medical histories, laboratory examinations, imaging examinations, inpatient complications, adverse outcomes, and treatment therapies in the hospital and at discharge, from the electronic health records. All laboratory testing and imaging examinations were carried out in line with Sichuan University West China Hospital's standard operating procedures..

The National Institutes of Health Stroke Scale (NIHSS) was used at admission and discharge to evaluate stroke-related neurological deficits. The range of NIHSS scores is 0 to 42, with higher scores indicating more severe neurological impairment [18]. Further, the A^2^DS^2^ score (score ranging from 0 to 10) was calculated according to age, dysphagia, male sex, atrial fibrillation, and stroke severity [19]. A^2^DS^2^ score can easily be completed on admission, and it is a widely validated screening tool for SAP in China, Denmark, the United Kingdom, Spain, and France.

### Malnutrition screening tools

Nutritional risk screening was performed by professionally trained researchers using GNRI, CONUT, and PNI. The GNRI, CONUT, and PNI scores were measured within 4 h after admission to collect blood samples for analysis.

The GNRI score was computed using the following formula: [20] 1.48 × serum albumin (g/L) + 41.7 × (current weight in kilograms/ideal weight). The ideal weight was calculated using the Lorenz formula: height (cm)—100—[height (cm)—150]/4 for men and height (cm) – 100—[height (cm)-150]/2.5 for women. When the current weight exceeded the ideal body weight, we set (current weight in kilograms/ideal weight) as 1. The patients were then classified into three groups based on the GNRI score as follows: absent (> 98), mild (92–98), and moderate-severe (< 92).

The nutritional status of the patients was evaluated using CONUT [21] based on the amounts of serum albumin, lymphocytes, and total cholesterol. According to the score, the patients were categorized into three groups: absent malnutrition (0–1), mild malnutrition (2–4), and moderate to severe malnutrition (5–12) [17, 21].

The PNI score was computed as 10 × serum albumin (g/dL) + 0.005 × lymphocyte count (per mm^3^). A score of > 45 indicated the absence of malnutrition, while scores of 40–45 and < 40 indicated moderate and severe malnutrition, respectively [22, 23].

### Outcomes

In this study, SAP incident was the main outcome. According to the modified Centers for Disease Control and Prevention criteria, SAP diagnosis was assessed within 7 days after stroke by physicians, and provide necessary treatment assignment for confirmed patients [24]. SAP diagnosis was determined and collected by electronic medical records, it was further reviewed and validated by research committee. The median time of SAP diagnosis was 4 (2–6) days after admission.

### Statistical analysis

Missing data of covariates (less than 2%) were imputed by multiple imputation analysis. Normally distributed continuous variables were represented by means ± standard deviations, while non-normally distributed continuous variables were represented by medians with interquartile ranges. Categorical variables are represented as frequencies and percentages. The Kruskal–Wallis H test was used to compare normally distributed variables, whereas one-way analysis was used to evaluate non-normally distributed patient characteristics. For the purpose of comparing categorical variables, either Fisher's exact test or the chi-square test was used.

The restricted cubic linear splines of the PNI, CONUT, and GNRI scores with three evenly spaced knots were utilized in order to investigate the associations between malnutrition scoring systems and adjusted odds ratios (ORs) for SAP outcomes. These restricted cubic linear splines were determined using the Harrell et al. method.[25]. An investigation of the connection between malnutrition and the possibility of developing SAP using logistic regression was carried out. Patients with absent malnutrition according to the PNI, CONUT, and GNRI were used as references. The model was adjusted according to age, sex, drinking, and smoking, body mass index (BMI), dysphagia, white blood cell count (WBC), and serum creatinine, diabetes, atrial fibrillation, hypertension, coronary heart disease, hyperlipidemia and NIHSS.

In the risk factor-adjusted logistic regression model, subgroup analyses were stratified by age (< 65 vs. ≥ 65 years), sex (male vs. female), atrial fibrillation (yes vs. no), smoking (yes vs. no), WBC count (≤ 7 × 10^9^/L vs. > 7 × 10^9^/L), and NIHSS score (≤ 7 vs. > 7). The interaction effects of these variables with the three malnutrition indices and SAP were calculated. The area under the receiver operating characteristic (AUROC) curves and continuous net reclassification improvement (NRI) were constructed to assess the predictive ability of the GNRI, CONUT, and PNI scores for SAP and to determine the additional prognostic value of the three malnutrition indices beyond A^2^DS^2^ via DeLong test [26]. Spearman correlation analysis was used to investigate the correlation between the malnutrition scoring systems and age, systemic inflammatory markers, and IS severity scores.

All *P* values were two-tailed, and the significance threshold was set at 0.05. All statistical analyses were conducted using SPSS version 26.0 (IBM Corp., Armonk, NY, USA) and R version 3.5.0. (R Foundation for Statistical Computing, Vienna, Austria).

## Results

### Baseline patient characteristics

A total of 915 participants with IS were enrolled, with an average age of 64.65 ± 14.06 years. Of these participants, 576 (63.0%) were men. Additionally, 294 (32.1%) patients developed SAP. The baseline characteristics of individuals with SAP and those without SAP are compared in Table 1. Participants who had SAP were older, had a higher prevalence of atrial fibrillation and coronary heart disease, and had a lower prevalence of complex hyperlipidemia. They also had higher dysphagia event, lower weight, BMI, hemoglobin, platelet count, lymphocyte count, albumin, and triglycerides, and higher admission temperature, systemic inflammation index (SII), WBC count, neutrophil count, D-dimer, fibrinogen, blood glucose, and blood urea nitrogen.

**Table 1:** Relationships between baseline clinical characteristics and stroke-associated pneumonia (SAP) in patients with ischemic stroke

Characteristics	Total (*n* = 915)	Non-SAP (*n* = 621)	SAP (*n* = 294)	*P*-value
**Demographic Variables**				
Age, years	64.65 ± 14.06	62.11 ± 13.54	69.48 ± 13.79	< 0.001
Males, n (%)	576 (63.0)	400 (64.4)	176 (59.9)	0.209
Smoking, n (%)	380 (41.5)	269 (43.3)	111 (37.8)	0.128
Drinking, n (%)	276 (30.2)	191 (30.8)	85 (28.9)	0.624
Hypertension, n (%)	542 (59.2)	247 (39.8)	126 (42.9)	0.416
Diabetes, n (%)	221 (24.2)	150 (24.2)	71 (24.1)	> 0.999
Hyperlipidemia, n (%)	85 (9.3)	68 (11.0)	17 (5.8)	0.017
Atrial fibrillation, n (%)	222 (24.3)	106 (17.1)	116 (39.5)	< 0.001
Coronary heart disease, n (%)	93 (10.2)	57 (9.2)	36 (12.2)	0.188
History of cancer, n (%)	43 (4.7)	23 (3.7)	20 (6.8)	0.057
**Physiological and Lab Variables**				
BMI, kg/m^2^ (IQR)	23.80 ± 3.29	24.10 ± 3.32	23.00 ± 3.09	< 0.001
Admission SBP, mmHg	145 ± 26	145 ± 26	145 ± 24	0.945
Admission DBP, mmHg	87 ± 16	88 ± 15	87 ± 17	0.433
Heart rate, beats/min	82 ± 23	81 ± 19	81 ± 24	0.638
Temperature, ℃	36.50 ± 0.29	36.47 ± 0.25	36.54 ± 0.37	0.001
Dysphagia, n (%)	80 (8.7)	26 (4.2)	54 (18.4)	< 0.001
Hemoglobin, g/L (IQR)	137 (125–148)	139 (127–150)	133 (120–144)	< 0.001
WBC, 10^9^/L (IQR)	7.12 (5.89–9.12)	6.76 (5.70–8.40)	8.42 (6.43–10.30)	< 0.001
Neutrophil count, 10^9^/L (IQR)	4.96 (3.71–7.19)	4.49 (3.47–6.26)	6.56 (4.61–8.42)	< 0.001
Platelet count, 10^9^/L (IQR)	176 (140–220)	180 (143–226)	169 (134–207)	0.010
Lymphocyte, 10^9^/L (IQR)	1.41 (1.00–1.86)	1.52 (1.12–1.96)	1.16 (0.83–1.58)	< 0.001
Albumin, g/L (IQR)	42.1 (39.6–44.5)	42.9 (40.5–44.9)	40.5 (37.4–43.0)	< 0.001
D-dimer, mg/L (IQR)	0.77 (0.34–2.15)	0.57 (0.28–1.18)	1.94 (0.91–4.74)	< 0.001
Fibrinogen, g/L (IQR)	2.86 (2.41–3.49)	2.82 (2.37–3.27)	3.10 (2.48–3.86)	< 0.001
Blood glucose, mmol/L (IQR)	6.20 (5.70–6.51)	6.20 (5.70–6.60)	6.20 (5.70–6.51)	0.255
Creatinine, umol/L (IQR)	74 (63–87)	73 (63–85)	76 (63–93)	0.243
BUN, mmol/L (IQR)	5.6 (4.5–7.1)	6.0 (4.7–7.9)	5.5 (4.4–6.8)	< 0.001
Triglycerides, mmol/L (IQR)	1.27 (0.92–1.85)	1.37 (0.97–1.98)	1.12 (0.88–1.57)	< 0.001
Total cholesterol, mmol/L (IQR)	4.59 (3.81–5.43)	4.60 (3.83–5.48)	4.56 (3.76–5.36)	0.147
HDL, mmol/L (IQR)	1.20 (0.97–1.46)	2.41 (1.90–3.05)	2.35 (1.83–2.90)	0.237
LDL, mmol/L (IQR)	2.39 (1.88–3.03)	1.21 (0.98–1.45)	1.18 (0.95–1.47)	0.420
**Risk scores**				
NIHSS score (IQR)	5 (3–12)	4 (2–6)	14 (9–18)	< 0.001
A^2^DS^2^ score (IQR)	4 (2–5)	4 (1–4)	5 (4–7)	< 0.001
PSI score (IQR)	68 (55–84)	65 (52–77)	81 (62 -100)	< 0.001

Data were presented by n (%), median ± standard deviation, and interquartile range (IQR)

Abbreviations: *SBP *systolic blood pressure, *DBP *diastolic blood pressure, *BMI *body mass index, *COPD *chronic obstructive pulmonary disease, *WBC *white blood cell count, *BUN *Blood urea nitrogen, *HDL *high-density lipoprotein, *LDL *low-density lipoprotein, *NIHSS *National Institute of Health Stroke Scale, *CONUT *controlling nutritional status score, *GNRI *geriatric nutritional risk index, *PNI *prognostic nutritional index, PSI pneumonia severity index

### Prevalence of malnutrition

According to the PNI, CONUT, and GNRI scores, 193 (21.1%), 495 (54.1%), and 148 (16.1%) malnourished patients were screened, respectively. Of these patients, 140 (15.3%), 449 (49.1%), and 57 (6.2%) patients, respectively, were classified into mild malnutrition; moreover, 53 (5.7%), 46 (5.0%), and 91 (10.0%) patients, respectively, classified into moderate or severe malnutrition.

### Malnutrition scores and SAP

**Fig. 2:**
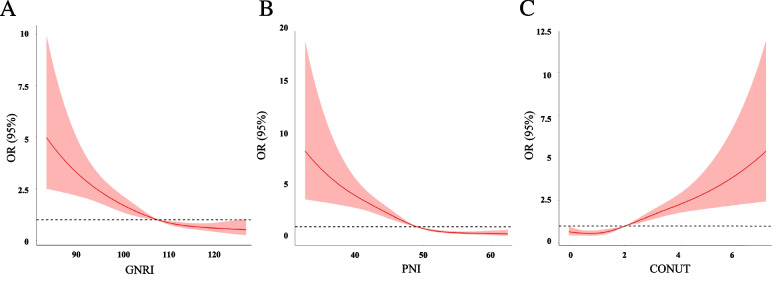
Adjusted hazard ratios (95% CI) of SAP outcome by linear splines of (**A**) PNI, (**B**) CONUT, and (**C**) GNRI with three same spacing knots. The solid line indicates the point estimate, and the shaded area is the 95% CI. Models were adjusted by age, sex, drinking, and smoking, body mass index (BMI), dysphagia, white blood cell count (WBC), and serum creatinine, diabetes, atrial fibrillation, hypertension, coronary heart disease, hyperlipidemia and NIHSS. Abbreviation: CONUT, controlling nutritional status score; GNRI, geriatric nutritional risk index; PNI, prognostic nutritional index; SAP, stroke-associated pneumonia

Patients (moderate and severe vs. mild vs. absent) with worse malnutrition risk had a higher incidence of SAP (PNI, 73.6% vs. 54.3% vs. 24.8%, *P* < 0.001; CONUT, 76.1% vs. 37.6% vs. 21.4%, *P* < 0.001; GNRI, 61.4% vs. 51.6% vs. 27.6%, *P* < 0.001; Supplementary Fig. 1). Restricted cubic spline analyses demonstrated continuous relationships between each malnutrition score and the adjusted ORs for SAP (Fig. 2). After adjusting for demographic variables, physical examination, laboratory testing, and chronic medical conditions in the logistic regression model, patients with moderate or severe malnutrition risk according to PNI (OR = 4.071, 95% CI 1.853–8.943, *P* < 0.001), CONUT (OR = 6.006, 95% CI 2.438–14.795, *P* < 0.001), and GNRI (OR = 2.556, 95% CI 1.322–4.943 *P* = 0.005) had a significantly higher SAP risk than patients with normal nutrition (Table 2).

**Table 2:** Logistic regression analysis regarding correlations between three malnutrition indexes and stroke-associated pneumonia

Indexes	Incidence of SAP (%)	Model 1		Model 2		Model 3	
		OR (95% CI)	*P*-value	OR (95% CI)	*P*-value	OR (95% CI)	*P*-value
**PNI**							
Absent	179/722 (24.8)	REF	-	REF	-	REF	-
Mild risk	76/140 (54.3)	3.602 (2.481–5.231)	< 0.001	2.666 (1.787–3.978)	< 0.001	2.339 (1.437–3.806)	0.001
Moderate and severe risk	39/53 (73.6)	8.451 (4.485–15.924)	< 0.001	6.615 (3.383–12.932)	< 0.001	4.071 (1.853–8.943)	< 0.001
**CONUT**							
Absent	90/420 (21.4)	REF	-	REF	-	REF	-
Mild risk	169/449 (37.6)	2.213 (1.537–2.991)	< 0.001	1.909 (1.384–2.634)	< 0.001	1.636 (1.113–2.406)	0.012
Moderate and severe risk	35/46 (76.1)	11.667 (5.699–23.884)	< 0.001	9.658(4.534–20.574)	< 0.001	6.006 (2.438–14.795)	< 0.001
**GNRI**							
Absent	212/767 (27.6)	REF	-	REF	-	REF	-
Mild risk	47/91 (51.6)	2.796 (1.800–4.344)	< 0.001	1.923 (1.415–4.997)	0.012	1.709 (1.006–2.904)	0.048
Moderate and severe risk	35/57 (61.4)	4.165 (2.388–7.264)	< 0.001	2.659 (1.415–4.997)	0.002	2.556 (1.322–4.943)	0.005

Model 1:is univariable Logistic regression analysis

Model 2: adjusted by model 1 plus adjusted by age, sex, BMI, smoking, drinking, dysphagia

Model 3: adjusted by model 2 plus hypertension, diabetes, hyperlipidemia, coronary heart disease, atrial fibrillation, white blood cell, creatinine, NIHSS

Abbreviations: *CONUT *controlling nutritional status score, *GNRI *geriatric nutritional risk index, *PNI *prognostic nutritional index, *OR *odds ratio, *CI *confidence interval, *BMI *body mass index, *HR *hazard ratio, *REF *reference

In addition, patients with higher severity of malnutrition, assessed by PNI, CONUT, and GNRI score, had higher risk of hospitalization mortality (Supplementary Fig. 2); while only PNI, CONUT were associated with longer length of stay (Supplementary Fig. 3).

### Predictive value of malnutrition for SAP

ROC curve analysis indicated the AUROCs of PNI, CONUT, and GNRI for SAP were 0.705 (95% CI: 0.670–0.740, *P* < 0.001), 0.643 (95% CI: 0.609–0.683, *P* < 0.001), and 0.646 (95% CI: 0.609–0.683, *P* < 0.001), respectively. The three malnutrition indices were inferior to A^2^DS^2^ (AUROC = 0.758; 95% CI: 0.724–0.791, *P* < 0.001). However, A^2^DS^2^ combined with PNI, CONUT, and GNRI achieved higher AUROCs (0.792, 0.774, and 0.775, respectively) than those achieved by A^2^DS^2^ alone (*P* < 0.05). The three malnutrition indexes achieved additional predictive values for SAP beyond the A^2^DS^2^ according to the NRI analysis (PNI: NRI = 0.064, 95% CI 0.022–0.108, *P* < 0.001; CONUT: NRI = 0.041, 95% CI 0.010–0.075, *P* < 0.001; GNRI: NRI = 0.049, 95% CI 0.013–0.084, *P* < 0.001) (Table 3).

**Table 3:** Predictive value of three malnutrition indexes and A^2^DS^2^ for stroke-associated pneumonia

Variables	AUROC (95% CI)	*P* for AUROC	*P* for AUROC comparison	NRI (95% CI)	*P* for NRI
A^2^DS^2^	0.758 (0.724, 0.791)	< 0.001	Ref	Ref	-
PNI	0.705 (0.670, 0.740)	< 0.001	< 0.001	-0.142 (-0.201, -0.077)	< 0.001
A^2^DS^2^ plus PNI	0.792 (0.724, 0.791)	< 0.001	< 0.001	0.064 (0.022, 0.108)	< 0.001
CONUT	0.643 (0.605, 0.682)	< 0.001	< 0.001	-0.185 (-0.258, -0.113)	< 0.001
A^2^DS^2^ plus CONUT	0.774 (0.741, 0.807)	< 0.001	0.032	0.041 (0.010, 0.075)	0.001
GNRI	0.646 (0.609, 0.683)	< 0.001	< 0.001	-0.174 (-0.259, -0.097)	< 0.001
A^2^DS^2^ plus GNRI	0.775 (0.742, 0.808)	< 0.001	0.003	0.049 (0.013. 0.084)	< 0.001

Abbreviations: *CONUT *controlling nutritional status score, *GNRI *geriatric nutritional risk index, *PNI *prognostic nutritional index, *CI *confidence interval, *AUROC *area under the receiver operating characteristic curve, *A*^*2*^*DS*^*2*^ consisted of age, atrial fibrillation, dysphagia, sex, stroke severity, *NRI *net reclassification improvement

### Subgroup analysis

**Fig. 3:**
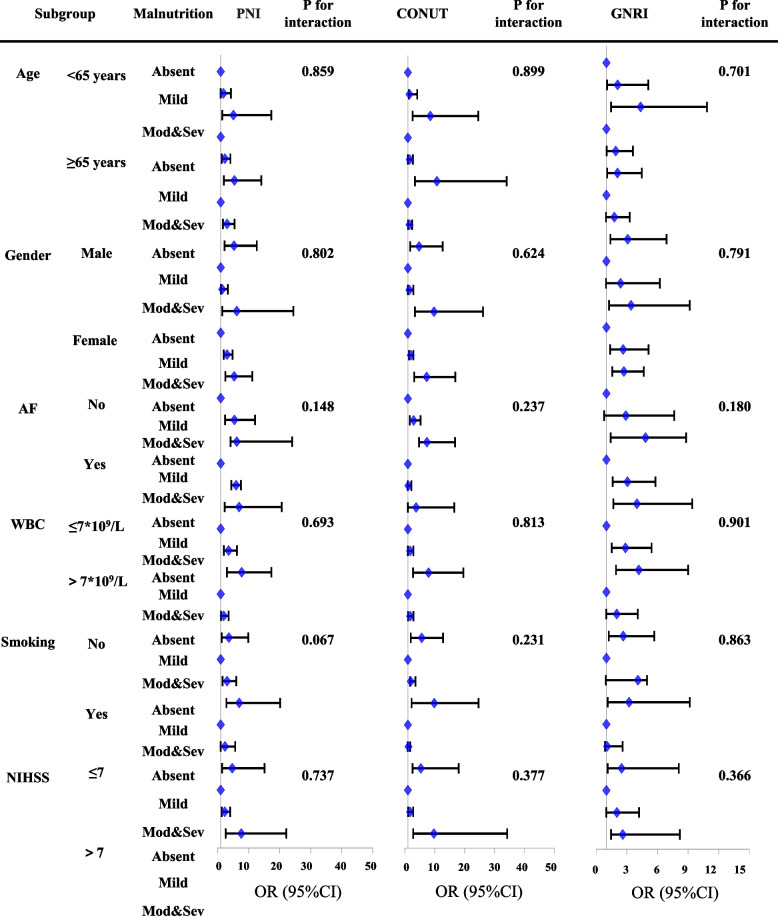
Odds ratio of SAI for different nutritional risk groups of PNI, CONUT and GNRI in demographic and clinical subgroups. The absent of malnutrition group was used as reference in Logistic regression models adjusted for age, sex, drinking, and smoking, body mass index (BMI), dysphagia, white blood cell count (WBC), and serum creatinine, diabetes, atrial fibrillation, hypertension, coronary heart disease, hyperlipidemia and NIHSS. Abbreviation: CONUT, controlling nutritional status score; GNRI, geriatric nutritional risk index; PNI, prognostic nutritional index; SAI, stroke-associated pneumonia; AF, atrial fibrillation; WBC, white blood cell; NIHSS, National Institute of Health Stroke Scale; OR, odds ratio; CI, confidence interval

Stratified subgroup analysis based on age, sex, BMI, treatment, cardioembolic stroke, and NIHSS score was performed. The results showed that the associations between SAP and malnutrition were consistent in different subgroup **(**Fig. 3).

### Malnutrition scores and the severity of IS

Spearman correlation analysis showed that the scoring systems such PNI, CONUT or GNRI, were associated with age, systemic inflammatory markers (SII, WBC count, NLR, neutrophil count, lymphocyte count, and monocyte count), severity of IS (NIHSS, and A^2^DS^2^), and the pneumonia severity index. The detailed results are shown in Supplementary Fig. 4.

## Discussion

Using various nutritional screening techniques, we demonstrated that malnutrition was prevalent among patients with IS in our study. Moreover, a greater severity of malnutrition was related to the incidence of SAP risk in individuals with IS, and after adjusting for confounding factors; a poor malnutrition index was an independent predictor of SAP, and this association consistent in different age, gender, and severity of IS. Furthermore, malnutrition could provide additional information on predicting the occurrence of SAP outcome, and adding malnutrition indices to A^2^DS^2^ enhanced the prediction of SAP. Therefore, our results indicate that malnutrition indices are useful tools for identifying high-risk SAP patients during early admission, and nutritional management may play a critical role in preventing SAP.

SAP is a frequent medical consequence after IS, and is mainly affected by the severity of IS [6, 27]. Several scoring systems that depend on severity-associated indices have been developed for evaluating the risk of SAP [2, 3, 13, 19]. Recent advancements in the prevention of SAP have been limited; clinical randomized controlled trials evaluating the use of preventive antibiotics for SAP have not yielded positive results, despite successes in animal research [28–30]. Comprehensive SAP risk control (CSRC) may be an efficient precaution [31, 32]; however, it is difficult to achieve the ideal preventive effect for CSRC based only on the severity of IS; thus, a novel dimension of CSRC identified should be identified.

The incidence of malnutrition in IS patients is considerable and varies significantly from 6% to 62.5% according to the nutritional screening measures used [16, 17]. According to the findings of several research, the majority of people who have IS also suffer from malnutrition, as assessed by the CONUT, GNRI, Malnutrition Universal Screening Tool, ranged widely from 7.1% to 18.2%, 13.5% to 31.8%, and 21.5% to 36%, respectively. Additionally, the incidences of IS patients at danger of malnutrition evaluated by Nutritional Risk Screening 2002 and the European Society of Clinical Nutrition and Metabolism-Diagnostic Criteria for Malnutrition (ESPEN-DCM) were 45.0% and 15.3%, respectively [14, 16, 17, 33–35]. In our study, 16.1% and 54.1% of individuals with IS were categorized as malnourished based on different grading methods. Although early screening tools may vary greatly due to differences in focus indicators, such as body weight, albumin, inflammation, and immune; malnutrition screening tools is effective to identify people who may have or develop to malnutrition. Therefore, malnutrition cannot be ignored in patients with IS, and patients screened for malnutrition (especially severe malnutrition) need more rigorous daily nutritional assessments and interventions.

Some randomized controlled trials and systematic reviews have indicated that early nutrition therapy could decrease the amount of systemic inflammation, improve immune function, and reduce the risk of stroke-associated infection [15]. Although the NRI only achieved a 4% to 6% improvement beyond A^2^DS^2^, this study provides a strong insight that clinicians should pay attention to the impact of malnutrition on SAP, and early malnutrition assessment may be of benefit to identify individuals with high risk of SAP from nutritional intervention.

Previous studies have indicated that malnutrition increases the danger of severe disability and mortality [16, 17]. Patients with IS who are hospitalized and undernourished have worse long-term survival, and increasing degrees of malnutrition are independently associated with an increased risk of mortality [17, 34, 36]. Studies have shown that severe malnutrition is connected with a 3.6 to 4.6-fold increase in the risk of long-term mortality among IS patients [16, 17, 33, 34, 36]. In addition, the malnutrition indices were slightly associated with poor functional outcomes [16, 33, 36]. CONUT, GNRI, and ESPEN-DCM scores at hospital were independently correlated with functional recovery at three months, but not at 12 months [16]. Furthermore, the functional outcome (modified Rankin scale ≥ 3) in IS patients with severe malnutrition was 2.5 to 10.1 times worse [16, 33, 36]. In our study, the malnutrition indices had obvious correlations with the scoring systems of IS severity, and the predictive values for SAP were maintained irrespective of the severity of IS in the subgroup analysis. Therefore, malnutrition may not only be a sign of severe IS in patients who are susceptible to SAP but may also be correlated with the severity of IS.

The components of malnutrition indices, such as inflammatory or nutritional indicators, may explain the association between malnutrition and SAP. Several studies have reported that biomarkers of inflammation (such as leukocyte count and its subtypes, NLR, C-reactive protein, procalcitonin, and interleukin-6) predicted SAP events in IS patients, and there was a strong association between NLR and pneumonia severity [1, 10–12]. In our study, the malnutrition scoring systems, CONUT, GNRI, and PNI, consisted of some inflammatory markers (serum albumin and lymphocyte count) and nutritional markers (total cholesterol, BMI), and were associated with inflammatory biomarkers and systemic inflammation score, suggesting that malnutrition may reflect high inflammatory and metabolic status, which contributes to the development of SAP. Lastly, severe malnutrition may be a surrogate marker for a larger number of risk factors for SAP and has been linked to age and other chronic metabolic diseases, which are also proven risk factors for SAP.

The present study had a few limitations. First, it was a multicenter retrospective cohort research done at stroke sites in the emergency departments of tertiary hospitals in China. Although the sample size was quite large, bias may have occurred in patient selection, and caution is required when evaluating the results of clinical nutritional assessments from different institutions. Second, we collected clinical data only at admission and discharge. The initial malnutrition indices of patients at admission were collected and could not be estimated at different time points. The dynamic fluctuation in nutritional scores assessed at different time points during hospitalization may be a better predictor of SAP. In addition, other risk factors and scoring systems related to SAP could not be continuously and dynamically evaluated. Third, patients with missing data (*n* = 82) to calculate PNI, CONUT, or GNRI scores were excluded, that may cause bias. Fourth, although patients were enrolled within 12 h of IS onset in this trial, blood sample collection was completed within 1 h of entry. This could still have introduced biases based on patient differences. Fifth, considering that it is a retrospective study, there may be potential confounding factors that have not been fully adjusted. Sixth, although we evaluated the predictive efficacy of the three nutrition screening measures, we did not compare them to other nutrition screening tools with more thorough nutrition assessments. Finally, we could not collect data on dietary components to examine the relationship between dietary constituents and nutritional status.

## Conclusion

Our findings revealed that malnutrition was frequent among IS patients and was connected with an increased danger of SAP, and early nutritional evaluation scoring tools could assist in identifying patients at a high risk for SAP. Therefore, malnutrition may become a possible intervention target for preventing SAP in patients with IS. However, more well-designed trials are necessary to prove the benefits of nutritional intervention in terms of SAP. 

### Supplementary Information

**Additional file1:Supplementary Fig. 1.** The incidence of SAP according to different nutritional risk groups of three malnutrition indexes (A) PNI, (B) CONUT, and (C) GNRI. CONUT, controlling nutritional status score, GNRI, geriatric nutritional risk index, PNI, prognostic nutritional index SAP, stroke-associated pneumonia. **Supplementary Fig. 2.** The incidence of all-cause mortality according to different nutritional risk groups of three malnutrition indexes (A) PNI, (B) CONUT, and (C) GNRI. Abbreviation: CONUT, controlling nutritional status score, GNRI, geriatric nutritional risk index, PNI, prognostic nutritional index. **Supplementary Fig. 3.** The length of stay according to different nutritional risk groups of three malnutrition indexes (A) PNI, (B) CONUT, and (C) GNRI. Abbreviation: CONUT, controlling nutritional status score, GNRI, geriatric nutritional risk index, PNI, prognostic nutritional index. **Supplementary Fig. 4.** Spearman correlation anaysis analyse the correlation of three malnutrition scoring systems and age, systemic inflammatory markers, the severity of IS, and the severity index of pneumonia. Abbreviation: SII, systemic inflammatory index, WBC, white blood cell count, NLR, neutrophil to lymphocyte ratio, PSI, pneumonia severity index.

## Data Availability

The data that support the findings of this study are available on request from the corresponding author.

## Abbreviations

AUROC: Area under the receiver operating characteristic curve
BMI: Body mass index
CI: Confidence interval
CONUT: Controlling Nutritional Status
CSRC: Comprehensive SAP risk control
ESPEN-DCM: European Society of Clinical Nutrition and Metabolism-Diagnostic Criteria for Malnutrition
GNRI: Geriatric Nutritional Risk Index
IS: Ischemic stroke
NIHSS: National Institutes of Health Stroke Scale
NLR: Neutrophil to lymphocyte ration
NRI: Net reclassification index
OR: Odds ratio
PNI: Prognostic Nutritional Index
REMISE: REtrospective Multicenter study for Ischemic Stroke Evaluation
ROC: Receiver operating characteristic
SAP: Stroke-associated pneumonia
SII: Systemic inflammation index
WBC: White blood cell count
