# Supplementary material for: Association between malnutrition and stroke-associated pneumonia in patients with ischemic stroke
Source: BMC Neurol. 2023 Aug 3;23:290. doi: 10.1186/s12883-023-03340-1 (PMC10399066; doi:10.1186/s12883-023-03340-1)
Supplement: Supplementary file 1 — Additional file1:Supplementary Fig. 1. The incidence of SAP according to different nutritional risk groups of three malnutrition indexes (A) PNI, (B) CONUT, and (C) GNRI. CONUT, controlling nutritional status score, GNRI, geriatric nutritional risk index, PNI, prognostic nutritional index SAP, stroke-associated pneumonia. Supplementary Fig. 2. The incidence of all-cause mortality according to different nutritional risk groups of three malnutrition indexes (A) PNI, (B) CONUT, and (C) GNRI. Abbreviation: CONUT, controlling nutritional status score, GNRI, geriatric nutritional risk index, PNI, prognostic nutritional index. Supplementary Fig. 3. The length of stay according to different nutritional risk groups of three malnutrition indexes (A) PNI, (B) CONUT, and (C) GNRI. Abbreviation: CONUT, controlling nutritional status score, GNRI, geriatric nutritional risk index, PNI, prognostic nutritional index. Supplementary Fig. 4. Spearman correlation anaysis analyse the correlation of three malnutrition scoring systems and age, systemic inflammatory markers, the severity of IS, and the severity index of pneumonia. Abbreviation: SII, systemic inflammatory index, WBC, white blood cell count, NLR, neutrophil to lymphocyte ratio, PSI, pneumonia severity index. [file 12883_2023_3340_MOESM1_ESM.pptx]

## Slide 1
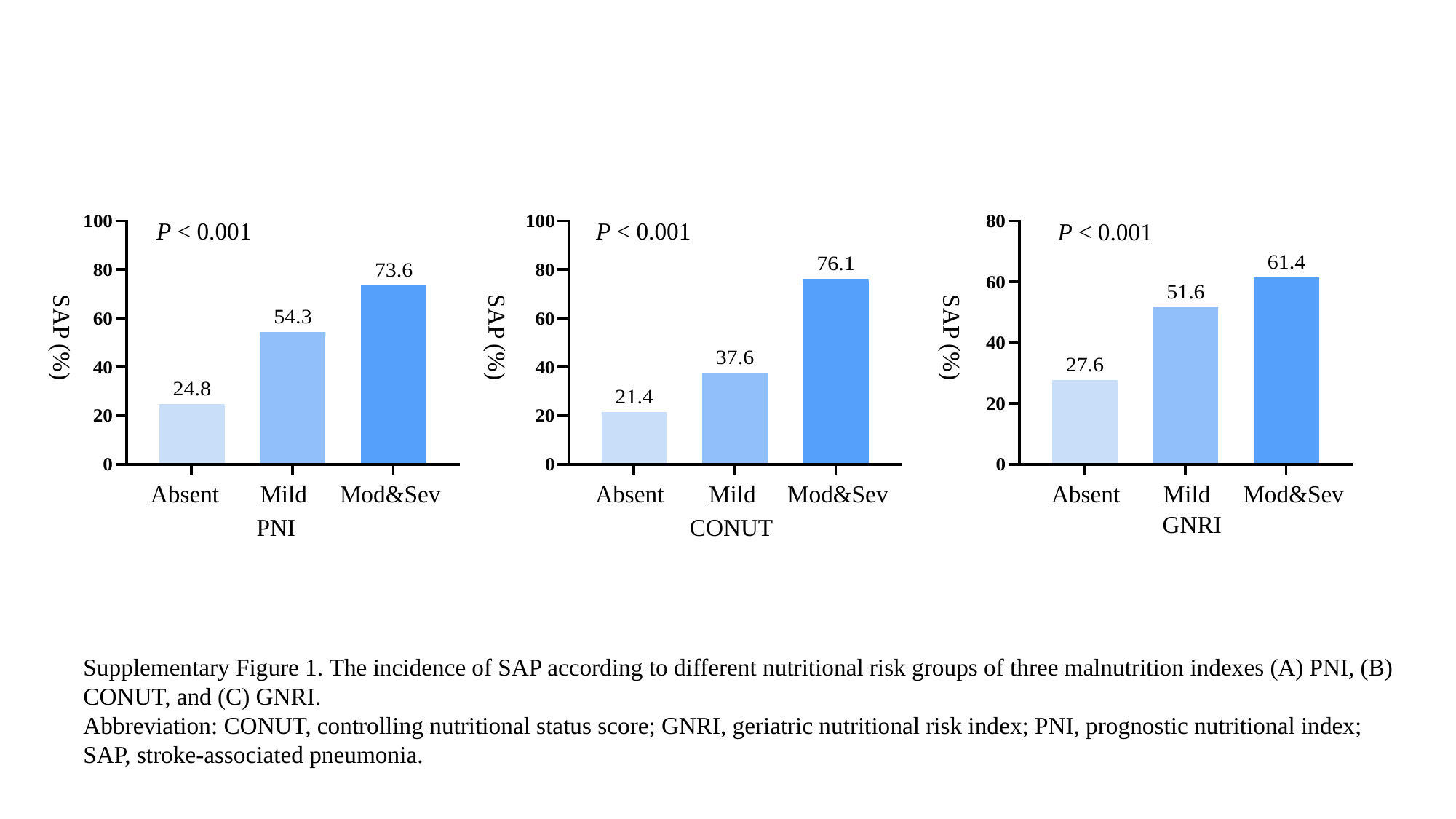

P < 0.001
P < 0.001
P < 0.001
SAP (%)
SAP (%)
SAP (%)
Absent
Mild
Mod&Sev
Absent
Mild
Mod&Sev
Absent
Mild
Mod&Sev
GNRI
PNI
CONUT
Supplementary Figure 1. The incidence of SAP according to different nutritional risk groups of three malnutrition indexes (A) PNI, (B) CONUT, and (C) GNRI.
Abbreviation: CONUT, controlling nutritional status score; GNRI, geriatric nutritional risk index; PNI, prognostic nutritional index; SAP, stroke-associated pneumonia.

## Slide 2
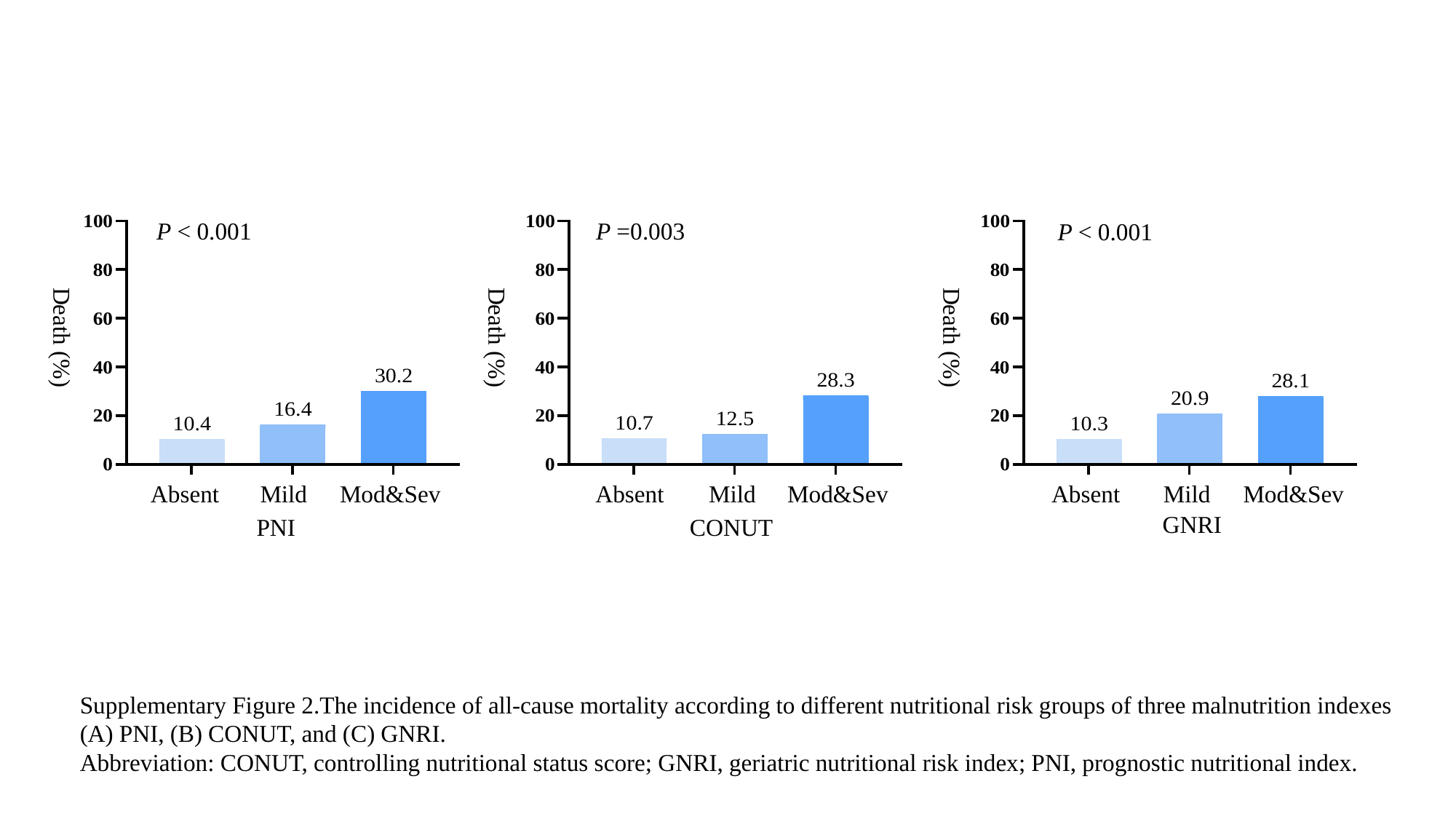

P < 0.001
P =0.003
P < 0.001
Death (%)
Death (%)
Death (%)
Absent
Mild
Mod&Sev
Absent
Mild
Mod&Sev
Absent
Mild
Mod&Sev
GNRI
PNI
CONUT
Supplementary Figure 2.The incidence of all-cause mortality according to different nutritional risk groups of three malnutrition indexes (A) PNI, (B) CONUT, and (C) GNRI.
Abbreviation: CONUT, controlling nutritional status score; GNRI, geriatric nutritional risk index; PNI, prognostic nutritional index.

## Slide 3
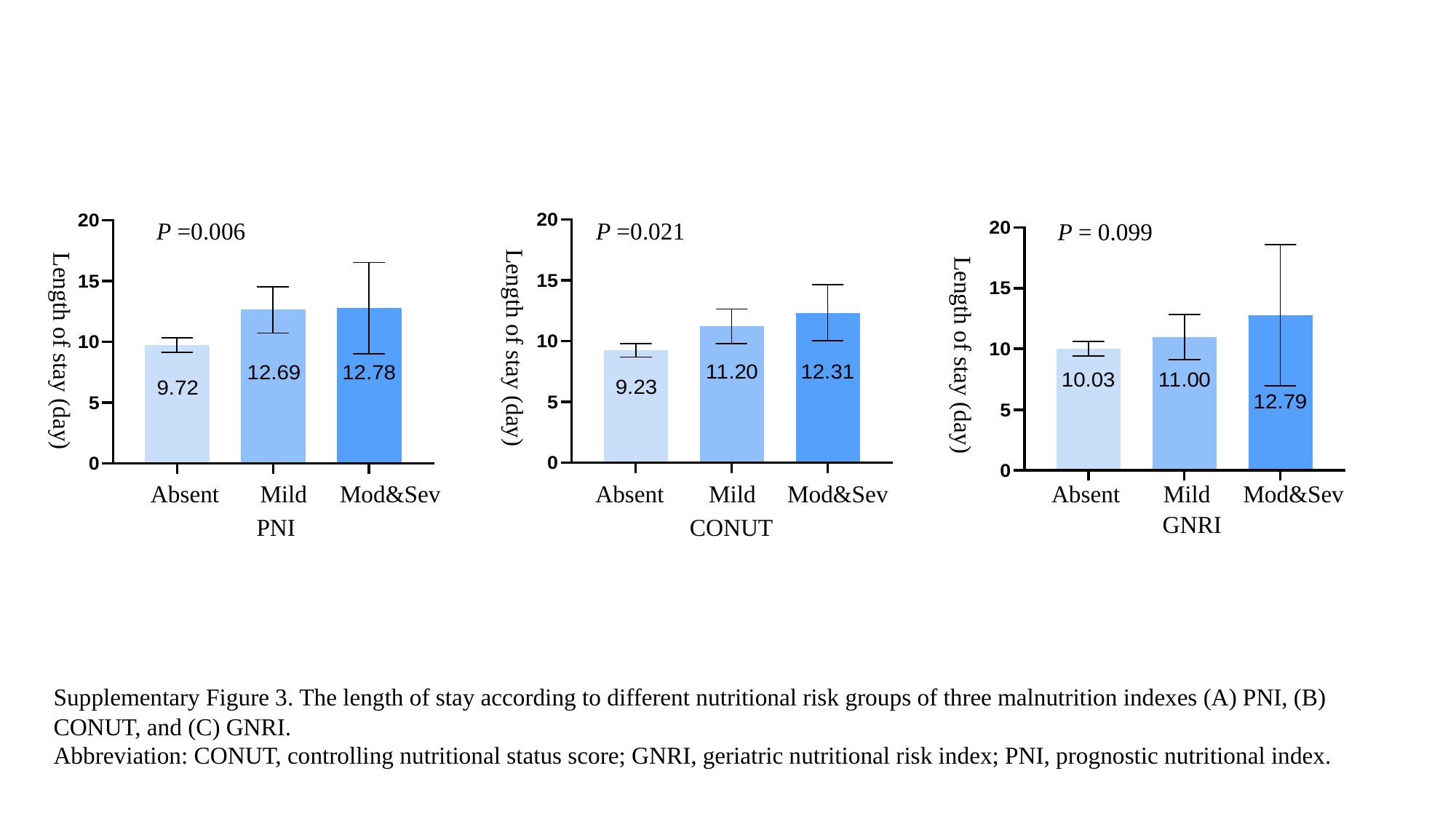

P =0.006
P =0.021
P = 0.099
Length of stay (day)
Length of stay (day)
Length of stay (day)
Absent
Mild
Mod&Sev
Absent
Mild
Mod&Sev
Absent
Mild
Mod&Sev
GNRI
PNI
CONUT
Supplementary Figure 3. The length of stay according to different nutritional risk groups of three malnutrition indexes (A) PNI, (B) CONUT, and (C) GNRI.
Abbreviation: CONUT, controlling nutritional status score; GNRI, geriatric nutritional risk index; PNI, prognostic nutritional index.

## Slide 4
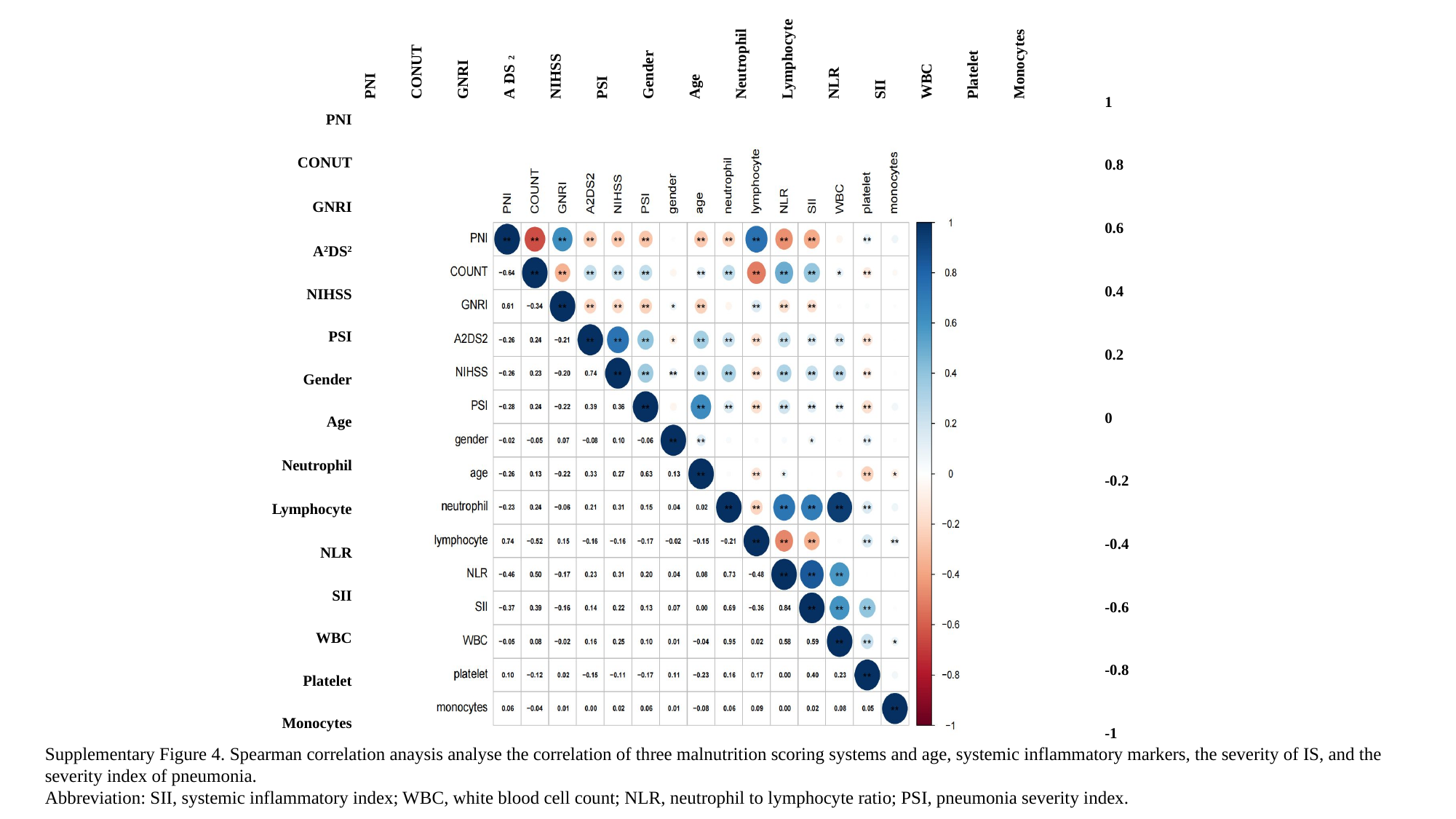

| PNI | CONUT | GNRI | A2DS2 | NIHSS | PSI | Gender | Age | Neutrophil | Lymphocyte | NLR | SII | WBC | Platelet | Monocytes |
| --- | --- | --- | --- | --- | --- | --- | --- | --- | --- | --- | --- | --- | --- | --- |
| 1 |
| --- |
| 0.8 |
| 0.6 |
| 0.4 |
| 0.2 |
| 0 |
| -0.2 |
| -0.4 |
| -0.6 |
| -0.8 |
| -1 |
| PNI |
| --- |
| CONUT |
| GNRI |
| A2DS2 |
| NIHSS |
| PSI |
| Gender |
| Age |
| Neutrophil |
| Lymphocyte |
| NLR |
| SII |
| WBC |
| Platelet |
| Monocytes |
Supplementary Figure 4. Spearman correlation anaysis analyse the correlation of three malnutrition scoring systems and age, systemic inflammatory markers, the severity of IS, and the severity index of pneumonia.
Abbreviation: SII, systemic inflammatory index; WBC, white blood cell count; NLR, neutrophil to lymphocyte ratio; PSI, pneumonia severity index.
